# Adaptive Multimodal Neuroimage Integration for Major Depression Disorder Detection

**DOI:** 10.3389/fninf.2022.856175

**Published:** 2022-04-29

**Authors:** Qianqian Wang, Long Li, Lishan Qiao, Mingxia Liu

**Affiliations:** ^1^School of Mathematics Science, Liaocheng University, Liaocheng, China; ^2^Taian Tumor Prevention and Treatment Hospital, Taian, China; ^3^Department of Radiology and BRIC, University of North Carolina at Chapel Hill, Chapel Hill, NC, United States

**Keywords:** major depressive disorder, resting-state functional MRI, structural MRI, feature adaptation, multimodal data fusion

## Abstract

Major depressive disorder (MDD) is one of the most common mental health disorders that can affect sleep, mood, appetite, and behavior of people. Multimodal neuroimaging data, such as functional and structural magnetic resonance imaging (MRI) scans, have been widely used in computer-aided detection of MDD. However, previous studies usually treat these two modalities separately, without considering their potentially complementary information. Even though a few studies propose integrating these two modalities, they usually suffer from significant inter-modality data heterogeneity. In this paper, we propose an adaptive multimodal neuroimage integration (AMNI) framework for automated MDD detection based on functional and structural MRIs. The AMNI framework consists of four major components: (1) a graph convolutional network to learn feature representations of functional connectivity networks derived from functional MRIs, (2) a convolutional neural network to learn features of T1-weighted structural MRIs, (3) a feature adaptation module to alleviate inter-modality difference, and (4) a feature fusion module to integrate feature representations extracted from two modalities for classification. To the best of our knowledge, this is among the first attempts to adaptively integrate functional and structural MRIs for neuroimaging-based MDD analysis by explicitly alleviating inter-modality heterogeneity. Extensive evaluations are performed on 533 subjects with resting-state functional MRI and T1-weighted MRI, with results suggesting the efficacy of the proposed method.

## 1. Introduction

Major depressive disorder (MDD) is one of the most common mental health disorders, affecting as many as 300 million people annually (Organization et al., 2017). This disease is generally characterized by depressed mood, diminished interests, and impaired cognitive function (Alexopoulos, 2005; Pizzagalli et al., 2008; Otte et al., 2016). Despite decades of research in basic science, clinical neuroscience and psychiatry, the pathological, and biological mechanisms of major depression remain unclear (Holtzheimer III and Nemeroff, 2006). The traditional diagnosis of MDD mainly depends on criteria from the diagnostic and statistical manual of mental disorders (DSM) and treatment response (Papakostas, 2009), which could be subjective and susceptible. As a robust complement to clinical neurobehavior-based detection, computer-aided diagnosis based on data hold the promise of objective diagnosis and prognosis of mental disorders (Foti et al., 2014; Liu and Zhang, 2014; Bron et al., 2015; Shi et al., 2018; Zhang L. et al., 2020; Buch and Liston, 2021).

Multiple neuroimaging modalities, such as resting-state functional magnetic resonance imaging (rs-fMRI) and structural MRI (sMRI), can provide complementary information in discovering objective disease biomarkers, and have been increasingly employed in automated diagnosis of various brain disorders (Hinrichs et al., 2011). Resting-state fMRI helps capture large-scale abnormality or dysfunction on functional connectivity network (FCN) by measuring bold-oxygen-level-dependent (BOLD) signals of subjects (Van Den Heuvel and Pol, 2010; Wang et al., 2019; Zhang Y. et al., 2020; Sun et al., 2021), and thus, can measure hemodynamic response related to neural activity in the brain dynamically. Structural MRI provides relatively high-resolution structural information of the brain, enabling us to study pathological changes in different brain tissues, such as gray matter (GM), white matter (WM), and cerebrospinal fluid (CSF) (Cuadra et al., 2005). It is critical to integrate rs-fMRI and sMRI data to facilitate automated diagnosis of MDD and related disorders.

Existing neuroimaging-based MDD studies usually focus on discovering structural or functional imaging biomarkers, by employing various machine learning approaches such as support vector machines (SVM), Gaussian process classifier (GPC), linear discriminant analysis (LDA), and deep neural networks (Sato et al., 2015; Bürger et al., 2017; Rubin-Falcone et al., 2018; Li et al., 2021). However, these methods generally ignore the potentially complementary information conveyed by functional and structural MRIs. Several recent studies propose to employ functional and structural MRIs for MDD analysis, but they usually suffer from significant inter-modality data discrepancy (Fu et al., 2015; Maglanoc et al., 2020; Ge et al., 2021).

**Figure 1:**
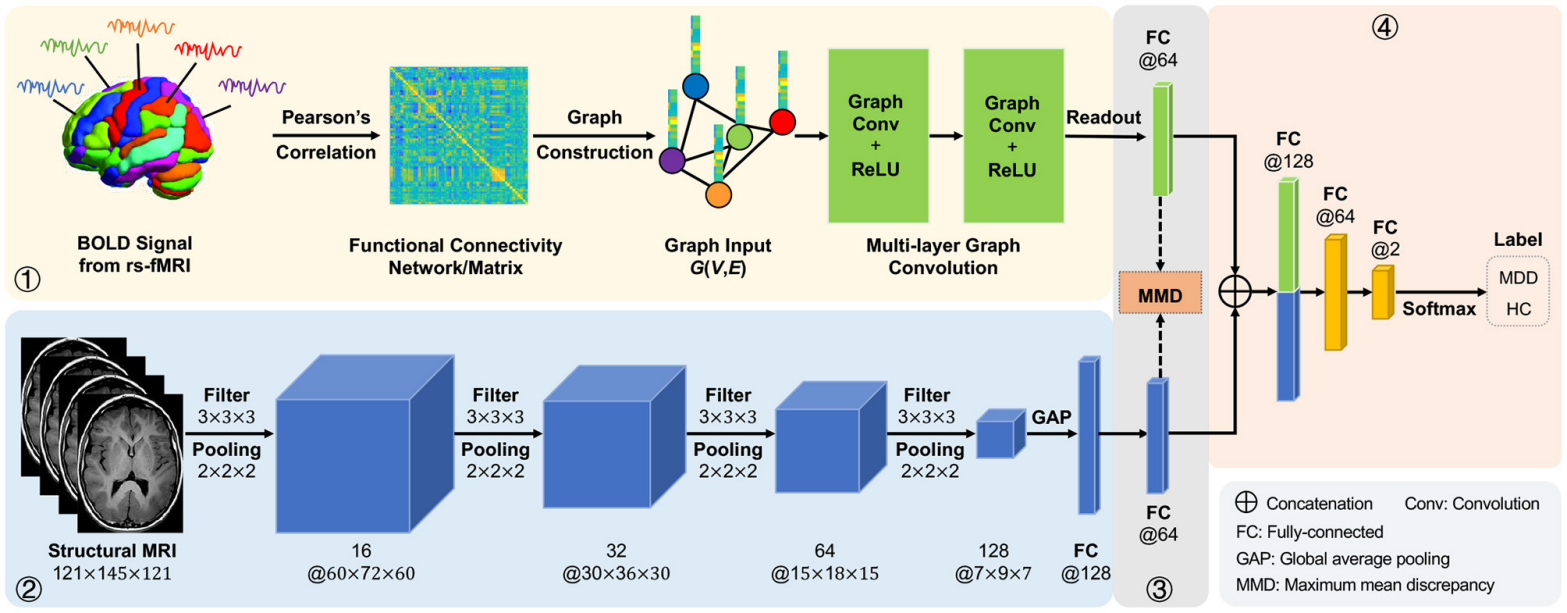
Illustration of the proposed adaptive multimodal neuroimage integration (AMNI) framework, including (1) a graph convolutional network (GCN) for extracting features of functional connectivity networks derived from resting-state functional MRI (rs-fMRI) data, (2) a convolutional neural network (CNN) for extracting features of T1-weighted structural MRI (sMRI) data, (3) a feature adaptation module for alleviating inter-modality difference by minimizing a cross-modal maximum mean discrepancy (MMD) loss, and (4) a feature fusion module for integrating sMRI and fMRI features for classification. MDD, major depressive disorder; HC, healthy control.

In this article, we propose an adaptive multimodal neuroimage integration (**AMNI**) framework for automated MDD detection using functional and structural MRI data. As shown in Figure 1, the proposed AMNI consists of four major components: (1) a *graph convolutional network* (GCN) for extracting feature representations of functional connectivity networks derived from rs-fMRI scans; (2) a *convolutional neural network* (CNN) for extracting features representations of T1-weighted sMRI scans; (3) a *feature adaptation module* for alleviating inter-modality difference by minimizing a cross-modal maximum mean discrepancy (MMD) loss; and (4) a *feature fusion module* for integrating features of two modalities for classification (*via* Softmax). Experimental results on 533 subjects from the REST-meta-MDD Consortium (Yan et al., 2019) demonstrate the effectiveness of AMNI in MDD detection.

The major contributions of this work are summarized below:

- An adaptive integration framework is developed to fuse functional and structural MRIs for automated MDD diagnosis by taking advantage of the complementary information of the two modalities. This is different from previous approaches that focus on only discovering structural or functional imaging biomarkers for MDD analysis.
- A feature adaptation strategy is designed to explicitly reduce the inter-modality difference by minimizing a cross-modal maximum mean discrepancy loss to re-calibrate features extracted from two heterogeneous modalities.
- Extensive experiments on 533 subjects with rs-fMRI and sMRI scans have been performed to validate the effectiveness of the proposed method in MDD detection.

The rest of this article is organized as follows. In Section 2, we briefly review the most relevant studies. In Section 3, we first introduce the materials and then present the proposed method as well as implementation details. In Section 4, we introduce the experimental settings and report the experimental results. In Section 5, we investigate the effect of several key components in the proposed method and discuss limitations as well as possible future research directions. We finally conclude this article in Section 6.

## 2. Related Work

In this section, we briefly introduce the most relevant studies on structural and functional brain MRI analysis, as well as multimodal neuroimaging-based diagnosis of brain disorders.

### 2.1. Brain Structural MR Imaging Analysis

Currently, MRI is the most sensitive imaging test of the brain in routine clinical practice. Structural MRIs can non-invasively capture the internal brain structure and atrophy, assisting us to understand the brain anatomical changes caused by various mental disorders. Conventional sMRI-based MDD analysis is usually performed manually by human beings *via* visual assessment (Scheltens et al., 1992), which could be subjective and susceptible. To this end, many machine learning methods (Gao et al., 2018), such as support vector machines (SVM), Gaussian process classifier (GPC), and linear discriminant analysis (LDA), have been used for automated MRI-based MDD diagnosis. However, these methods generally rely on handcrafted MRI features and these features may be suboptimal for subsequent analysis, thus significantly limiting their practical utility.

In recent years, deep learning methods such as convolutional neural networks (CNNs) have been widely used in the fields of computer vision and medical image analysis (Yue-Hei Ng et al., 2015; Chen et al., 2016; Zhang L. et al., 2020). As a special type of multi-layer neural network, CNN is capable of automatic feature learning, which eliminates the subjectivity in extracting and selecting informative features for specific tasks (Lee et al., 2017). Based on the LeNet5 network, Sarraf and Tofighi (2016) presented a 2D convolutional neural network that could classify sMRI scan slices for Alzheimer's disease diagnosis. With the development of high-performance computing resources, Hosseini-Asl et al. (2016) developed a deep neural network that used 3D convolution layers to extract features of 3D medical images for Alzheimer's disease diagnosis. Chakraborty et al. (2020) developed a 3D CNN architecture for learning intricate patterns in MRI scans for Parkinson's disease diagnosis. Compared with 2D convolution, 3D convolution on the entire MR image is able to capture the rich spatial information, which is essential for disease classification.

### 2.2. Brain Functional MR Imaging Analysis

Existing studies have revealed that fMRI can capture large-scale abnormality or dysfunction on functional connectivity networks by measuring the blood-oxygen-level in the brain (Van Den Heuvel and Pol, 2010; Zhang et al., 2019). With fMRI data, we usually construct a functional connectivity network for representing each subject, where each node represents a specific brain region-of-interest (ROI) and each edge denotes the pairwise relationship between ROIs (Honey et al., 2009; Dvornek et al., 2017). By capturing the dependencies between BOLD signals of paired ROIs, functional connectivity networks (FCNs) have been widely used to identify potential neuroimaging biomarkers for mental disorder analysis. Previous studies often extract handcrafted FCN features (e.g., clustering coefficient and node degree) to build prediction/classification models (Guo et al., 2021; Zhang et al., 2021), but the definition of the optimal FCN features highly relies on expert knowledge, so it is often subjective. Extracting effective feature representations of functional connectivity networks is essential for subsequent analysis.

Recent studies have shown that spectral graph convolutional networks (GCNs) are effective in learning representations of brain functional connectivity networks, where each FCN is treated as a graph (Bruna et al., 2013; Parisot et al., 2018; Bai et al., 2020; Yao et al., 2021). Motivated by breakthroughs of deep learning on grid data, people make efforts to extend CNN to graphs, giving rise to the spectral graph convolutional networks (GCNs) (Bruna et al., 2013). Recent studies have shown that GCNs are effective in learning representations of brain functional connectivity networks compared to traditional machine learning algorithms. For example, Parisot et al. (2018) proposed a GCN-based method for group-level population diagnosis that exploited the concept of spectral graph convolutions. Yao et al. (2021) presented a mutual multi-scale triplet GCN model to extract multi-scale feature representations of brain functional connectivity networks. Bai et al. (2020) developed a backtrackless aligned-spatial GCN model to transitively align vertices between graphs and learn effective features for graph classification. Compared with traditional CNN with Euclidean data, GCN generalizes convolution operations to non-Euclidean data, and helps mine topological information of brain connectivity networks.

### 2.3. Multimodal Neuroimaging-Based Brain Disease Diagnosis

Previous studies have been shown that multimodal neuroimaging data can provide complementary information of individual subjects to improve the performance of computer-aided disease diagnosis (Sui et al., 2013; Calhoun and Sui, 2016; Maglanoc et al., 2020; Guan and Liu, 2021). For example, Sui et al. (2013) developed a machine learning model to enable fusion of three or more multimodal datasets based on multi-set canonical correlation analysis and joint independent component analysis algorithms. Maglanoc et al. (2020) used linked independent component analysis to fuse structural and functional MRI features for depression diagnosis. Even though previous studies have yielded promising performance, they often extract sMRI and fMRI features manually, which requires domain-specific knowledge (Shen et al., 2017). Several deep learning models of multimodal medical image fusion are proposed to employ multimodal neuroimaging data for brain disease diagnosis (Rajalingam and Priya, 2018). However, existing studies usually focus on combining feature representation of multiple modalities and ignore significant inter-modality heterogeneity (Huang et al., 2019). To this end, we propose an adaptive multimodal neuroimage integration (AMNI) framework for automated MDD diagnosis based on resting-state functional MRI and T1-weighted structural MRI data. The proposed method can not only extract high-level feature representations of structural and functional data *via* CNN and GCN, respectively, but also alleviate the heterogeneity between modalities with the help of a unique feature adaptation module.

## 3. Materials and Methods

In this section, we first introduce the materials and image pre-processing method used in this work, and then present the proposed method and implementation details.

### 3.1. Materials

#### 3.1.1. Data Acquisition

Resting-state fMRI and T1-weighted structural MRI data were acquired from 282 MDD subjects and 251 healthy controls (HCs) recruited from the Southwest University, an imaging site of the REST-meta-MDD consortium (Yan et al., 2019). Resting-state fMRI were acquired through a Siemens scanner with the following parameters: repetition time (TP) = 2, 000 *ms*, echo time (TE) = 30 *ms*, flip angle = 90*·*, slice thickness = 3.0 *mm*, gap = 1.0 *ms*, time point = 242, voxel size = 3.44 × 3.44 × 4.00 *mm*^3^. More detailed information can be found online^1^. The demographic and clinical information of these studied subjects is summarized in Table 1.

**Table 1:** Demographic and clinical information of subjects from Southwest University [a part of the REST-meta-MDD consortium (Yan et al., 2019)].

**Category**	**Gender**	**Age**	**Education**	**First period**	**On medication**	**Duration of illness**
MDD	99 M	38.7 ± 13.6	10.8 ± 3.6	209 (Y)/49 (N)	124 (Y)/125 (N)	50.0 ± 65.9
	183 F			24 (D)	33 (D)	35 (D)
HC	87 M	39.6 ± 15.8	13.0 ± 3.9	−	−	−
	164 F					

*Values are reported as Mean ± Standard deviation. M, Male; F, Female; Y, Yes; N, No; D, Lack of record*.

#### 3.1.2. Image Pre-processing

The resting-state fMRI and structural T1-weighted MRI scans were pre-processed using the Diffeomorphic Anatomical Registration Through Exponentiated Lie algebra (DPARSF) software (Yan and Zang, 2010) with a standardized protocol (Yan et al., 2016). For rs-fMRI data, we first discard the first 10 volumes the initial 10 volumes were discarded, and slice-timing correction was performed. Then, the time series of images for each subject were realigned using a six-parameter (rigid body) linear transformation. After realignment, individual T1-weighted images were co-registered to the mean functional image using a 6 degrees-of-freedom linear transformation without re-sampling and then segmented into gray matter (GM), white matter (WM) and cerebrospinal fluid (CSF). Finally, transformations from individual native space to MNI space were computed with the Diffeomorphic Anatomical Registration Through Exponentiated Lie algebra (DARTEL) tool (Ashburner, 2007). After that, the fMRI data were normalized with an EPI template in the MNI space, and resampled to the resolution of 3 × 3 × 3 *mm*^3^, followed by spatial smoothing using a 6 *mm* full width half maximum Gaussian kernel. Note that subjects with poor image quality or excessive head motion (mean framewise-displacement >0.2 *mm*) were excluded from analysis (Jenkinson et al., 2002). Finally, we extracted the mean rs-fMRI time series with band-pass filtering (0.01−0.1*Hz*) of a set of 112 pre-defined regions-of-interest (ROIs), including cortical and subcortical areas based on the Harvard-Oxford atlas. Each T1-weighted structural MR image was also segmented into three tissues (i.e., GM, WM, and CSF) and transformed into the MNI space with DARTEL tool (Ashburner, 2007), resulting in a 3D volume (size: 121 × 145 × 121). Here, we employ gray matter volume in the MNI space for representing the original sMRI.

### 3.2. Proposed Method

As illustrated in Figure 1, the proposed AMNI consists of four major components: (1) a GCN module to extract features from rs-fMRI, (2) a CNN module to extract features from T1-weighted sMRI, (3) a feature adaptation module to reduce inter-modality discrepancy, and (4) a feature fusion module for classification, with details introduced below.

#### 3.2.1. GCN for Functional MRI Feature Learning

Based on resting-state fMRI data, one usually constructs a functional connectivity matrix/network (FCN) for representing each subject, with each node representing a specific brain ROI and each edge denoting the pairwise functional connection/relationship between ROIs (Honey et al., 2009; Dvornek et al., 2017). That is, FCNs help capture the dependencies between BOLD signals of paired ROIs. Considering the fact that FCNs are non-Euclidean data, we treat each functional connectivity network as a specific graph and resort to spectral graph convolutional network (GCN) for FCN feature learning by capturing graph topology information. Previous studies have shown that GCN is effective in learning graph-level representations by gradually aggregating feature vectors of all nodes (Yao et al., 2019). In this work, we aim to learn graph-level representations based on node representations of input FCNs.

(**i**) **Graph Construction**. Denote *N* and *M* as the numbers of ROIs and time points, respectively, where *N* = 112 and *M* = 232 in this work. We assume that the rs-fMRI time-series data for a subject is $Y={\left(y_{1},\cdots \;,y_{N}\right)}^{T}\in R^{N\times M}$, where each element $y_{n}\in R^{M}$ (*n* = 1, ⋯ , *N*) denotes BOLD measurements of the *n*-th ROI at *M* successive time points.

As the simplest and most widely used method, Pearson correlation (PC) is usually used to construct functional connectivity networks from raw rs-fMRI time-series data. Denote $B=\left(b_{ij}\right)\in R^{N\times N}$ as the functional connectivity matrix based on the Pearson correlation algorithm. Each element *b*_*ij*_∈[−1, 1] in *B* represents the Pearson correlation coefficient between the *i*-th and *j*-th ROIs, defined as follows:


(1) $$\begin{array}{l} b_{ij}=\frac{{\left(y_{i}-{ȳ}_{i}\right)}^{T}\left(y_{j}-{ȳ}_{j}\right)}{\sqrt{{\left(y_{i}-{ȳ}_{i}\right)}^{T}\left(y_{i}-{ȳ}_{i}\right)}\sqrt{{\left(y_{j}-{ȳ}_{j}\right)}^{T}\left(y_{j}-{ȳ}_{j}\right)}} \end{array}$$

where ȳ_*i*_ and ȳ_*j*_ are the mean vector corresponding to $y_{i}\in R^{M}$ and $y_{j}\in R^{M}$, respectively, and *M* represents the length of time points of BOLD signals in each brain region.

For each subject, we regard each brain FCN as an undirected graph *G* = {*V, E*}, where *V* = {*v*_1_, ⋯  , *v*_*N*_} is a set of *N* nodes/ROIs and *b*_*ij*_∈*B* denotes the functional connectivity between a paired nodes *v*_*i*_ and *v*_*j*_. Since spectral GCNs work on adjacency matrices by updating and aggregating node features (Bruna et al., 2013), it is essential to generate such an adjacency matrix *A* and a node feature matrix *X* from each graph *G*.

To reduce the influence of noisy/redundant information, we propose to construct a K-Nearest Neighbor (KNN) graph based on each densely-connected functional connectivity matrix. Specifically, a KNN graph is generated by only keep the top k important edges according to their functional connectivity strength (i.e., PC coefficient) for each node. Then, the topology structure of the graph *G* can be described by adjacency matrix $A=\left(a_{ij}\right)\in {\left\{0,1\right\}}^{N\times N}$, where *a*_*ij*_ = 1 if there exists an edge between the *i*-th and the *j*-th ROIs, and *a*_*ij*_ = 0, otherwise. In addition, the node features are defined by the functional connection weights of edges connected to each node, i.e., corresponding to a specific row in the functional connectivity matrix. Thus, the node features of the graph *G* can be represented by the node feature matrix *X* = *B*.

(**ii**) **Graph Feature Learning**. In GCN models, the convolution operation on the graph is defined as the multiplication of filters and signals in the Fourier domain. Specifically, GCN model learns new node representations by calculating the weighted sum of feature vectors of central nodes and the neighboring nodes. Mathematically, the simplest spectral GCN layer (Kipf and Welling, 2016) can be formulated as:


(2) $$\begin{array}{l} H^{l+1}=f\left(H^{l},A\right)=\sigma \left(\tilde{A}H^{l}W^{l}\right) \end{array}$$

where *H*^*l*^ is the matrix of activations in the *l*-th layer, and *W*^*l*^ is a layer-specific trainable weight matrix.

In addition, $\tilde{A}=D^{-\frac{1}{2}}AD^{-\frac{1}{2}}$ is the normalized adjacency matrix with self loops, and σ(·) is an activation function, such as the *ReLU*(·) = *max*(0, ·). In addition, *D* is the diagonal degree matrix, with the *i*-th diagonal element defined as $d_{i}=\underset{i\neq j}{\sum }A_{ij}$.

In the GCN module in our AMNI framework, we stack two graph convolutional layers with the adjacency matrix *A* and node features matrix *X* as inputs. The output of this two-layer GCN module is calculated as:


(3) $$\begin{array}{l} Z=f\left(A,X\right)=ReLU\left(\tilde{A}ReLU\left(\tilde{A}XW^{\left(0\right)}\right)W^{1}\right) \end{array}$$

Note that the number of neurons in the two graph convolutional layers is set as 64 and 64, respectively.

Given that this is a graph classification task, we employ a simple graph pooling strategy (Lee et al., 2019) to generate graph-level FCN representations. To be specific, we employ both global average pooling and global max pooling that aggregate node features to generate new feature representations. The output feature of the graph pooling layer is as follows:


(4) $$\begin{array}{l} g_{F}=\frac{1}{N}\overset{N}{\underset{i=1}{\sum }}z_{i}||\overset{N}{\underset{i=1}{\max}}z_{i} \end{array}$$

where *N* is the number of ROIs, *z*_*i*_ is the feature vector of *i*-th ROI obtained by the graph convolution operation, and || denotes concatenation.

By stacking multiple graph convolution layers and graph pooling layers, GCN can learn higher-order node features from neighboring nodes. In addition, GCN propagates information on a graph structure and gradually aggregates the information of neighboring nodes, which allows us to effectively capture the complex dependencies among ROIs.

#### 3.2.2. CNN for Structural MRI Feature Learning

In recent years, convolutional neural networks (CNNs) have shown much predomination in image recognition and classification (Simonyan and Zisserman, 2014; He et al., 2016). Due to the 3D nature of structural MR images (sMRI), it is important to learn feature representations of all three dimensions from volumetric medical data. Considering that 3D convolutional kernels can encode richer spatial information, we adopt 3D CNN model to extract feature representations of T1-weighted MRIs.

In the AMNI framework, the CNN module consists of four convolution blocks and two fully-connected (FC) layers for local to global sMRI feature extraction. To be specific, each convolution block consists of one convolutional layer, one batch normalization layer, one activation function and one max pooling layer. To capture local patterns, 3D convolution is achieved by convolving a 3D kernel over 3D feature cubes. Formally, the *j*-th feature map in the *i*-th layer, denoted as *v*_*i, j*_, is given by


(5) $$\begin{array}{l} v_{i,j}=f\left(\left(W_{i,j}*V_{i-1}\right)+b_{i,j}\right) \end{array}$$

where *W*_*i, j*_ and *b*_*i, j*_ are the kernel weights and the bias for the *j*-th feature map, respectively, *V*_*i*−1_ are the sets of input feature maps connected to the current layer from the (*i*−1)*th* layer, * is the convolution operation, and *f* is the non-linear activation function. The size of each convolution filter is 3 × 3 × 3, and the numbers of convolution filters are set to 16, 32, 64, 128, respectively. In addition, max pooling is applied for each 2 × 2 × 2 region which reduces the spatial size of the feature maps and the number of parameters, and ReLU is used as the activation function. Meanwhile, batch normalization technique can promote faster convergence and better generalization of trained networks.

For the pooling layer, we use the Global Average Pooling (GAP) operation (Lin et al., 2013), which performs downsampling by computing the mean of the height, width, and depth dimensions of the input. The formula for GAP is as follows:


(6) $$\begin{array}{l} g_{j}=\frac{\overset{H}{\underset{h=1}{\sum }}\overset{W}{\underset{w=1}{\sum }}\overset{D}{\underset{d=1}{\sum }}v_{j}^{h,w,d}}{H\times W\times D} \end{array}$$

where $v_{j}^{h,w,d}$ is the value at position (*h, w, d*) of the *j*-th input feature map, *H*, *W*, and *D* are the height, width, and depth respectively and *g*_*j*_ is getting value of the *j*-th input feature map through GAP. Thus, the sMRI feature *g*_*S*_ generated by CNN is given by:


(7) $$\begin{array}{l} g_{S}={\left[g_{1},g_{2},\cdots \;,g_{c}\right]}^{T} \end{array}$$

where *c* is the number of input feature map. It can be seen that the GAP layer converts a 4D tensor to a 1-dimensional feature vector, thus significantly reducing the number of network parameters.

The two fully-connected layers have 128 and 64 neurons, respectively. To avoid overfitting, we employ the dropout technique (Srivastava et al., 2014), with a probability of 0.5 after each fully-connected layer. More detailed information about the CNN architecture can be found in Table 2.

**Table 2:** Architecture of the CNN module in the proposed AMINI framework.

** Layer**	**Kernel size**	**Stride**	**Output size**	**Feature volumes**
Input	–	–	121 ×145 ×121	1
C1	3 ×3 ×3	1	121 ×145 ×121	16
M1	2 ×2 ×2	2	60 ×72 ×60	16
C2	3 ×3 ×3	1	60 ×72 ×60	32
M2	2 ×2 ×2	2	30 ×36 ×30	32
C3	3 ×3 ×3	1	30 ×36 ×30	64
M3	2 ×2 ×2	2	15 ×18 ×15	64
C4	3 ×3 ×3	1	15 ×18 ×15	128
M4	2 ×2 ×2	2	7 ×9 ×7	128
GAP	–	–	1 ×1 ×1	128
FC	–		1 ×1 ×1	64

*Cn, the n-th convolutional layer; Mn, the n-th max pooling layer; GAP, global average pooling; FC, fully-connected layer*.

#### 3.2.3. Feature Adaptation Module

Due to the heterogeneous nature of multimodal data, it is necessary to reduce the discrepancy between feature representations of different modalities before feature fusion. Inspired by existing studies on domain adaptation (Tzeng et al., 2014), we employ a cross-modal loss based on maximum mean discrepancy (MMD) (Gretton et al., 2012) to re-calibrate channel-wise features extracted from sMRI and fMRI. Denote *G*_*F*_ and *G*_*S*_ as feature representations of fMRI and sMRI, respectively. The cross-modal MMD loss *L*_*M*_ is formulated as follows:


(8) $$\begin{array}{l} L_{M}=MMD(G_{F},G_{S})\\ \;=\;\left||\frac{1}{\left|G_{F}\right|}\sum_{g_{F}\in G_{F}}\phi (g_{F})-\frac{1}{\left|G_{S}\right|}\sum_{g_{S}\in G_{S}}\phi (g_{S})|\right| \end{array}$$

where ϕ(·) denotes the feature map associated with the kernel map, and *g*_*F*_ and *g*_*S*_ are elements in *G*_*F*_ and *G*_*S*_, respectively. During model training, the cross-modal MMD loss will be used as a regularization term to penalize heterogeneity of the features between the two modalities.

As shown in Figure 1, this cross-modal MMD loss is applied to features from two fully-connected layers in the proposed CNN and GCN modules. This would enable the feature adaptation module to learn shared and aligned information across modalities by minimizing the distribution difference between two feature representations.

#### 3.2.4. Feature Fusion Module

To enable our AMNI method to capture the complementary information provided by functional and structural MRIs, we also design a feature fusion module for classification/prediction.

Assuming that *F*_1_ and *F*_2_ are two feature representations obtained by feature adaptation module, we first concatenate them to obtain a new representation. The new representation *F* can be described as follows:


(9) $$\begin{array}{l} F={\left[F_{1}^{T},F_{2}^{T}\right]}^{T} \end{array}$$

After concatenation, the obtained new representation is fed to two fully-connected layers (with 64 and 2 neurons, respectively), and the learned features are further fed into a Softmax layer for classification.

During the training stage, we use the cross-entropy loss function to optimize the parameters in our AMINI model. The classification loss *L*_*C*_ is defined as:


(10) $$\begin{array}{l} L_{C}=-\frac{1}{N}\overset{N}{\underset{i=1}{\sum }}\left(y_{i}log\left(p_{i}\right)+\left(1-y_{i}\right)log\left(1-p_{i}\right)\right) \end{array}$$

where *N* is the number of samples, and *y*_*i*_ is the true label of the *i*-th sample, with 1 representing the sample being a MDD patient and 0 denoting the sample being a healthy control. In addition, *p* is the predicted probability that the sample belongs to the MDD category.

In our model, we aim to minimize not only the classification loss, but also the cross-modal loss to reduce the inter-modality difference. Hence, the total loss function *L* of the proposed AMNI is defined as follows:


(11) $$\begin{array}{l} L=L_{C}+\lambda L_{M} \end{array}$$

where λ is a hyperparameter to tune the contributions of two terms in Equation (11).

### 3.3. Implementation Details

We optimize the proposed AMNI model *via* the Adam (Kingma and Ba, 2014) algorithm, with the learning rate of 0.0001, weight decay rate of 0.0015, training epoch of 100, and mini-batch size of 16. The proposed model is implemented based on Pytorch (Paszke et al., 2017), and the model is trained by using a single GPU (NVIDIA Quadro RTX 6000 with 24 GB memory). The hyperparameter λ in Equation (11) is empirically set as 0.01. And we will experimentally investigate its influence in Section 5.

## 4. Experiments

In this section, we introduce experimental settings and several competing methods, present the experimental results, and visualize feature distributions of different methods.

### 4.1. Experimental Settings

We randomly select 80% samples as training data, and the remaining 20% samples are used as test data. To avoid bias introduced by random partition, we repeat the random partition procedure 10 times independently, and record the mean and standard deviation results. Eight metrics are used to evaluate the performance of different methods in the task of MDD detection (i.e., MDD vs. HC classification), including accuracy (ACC), sensitivity (SEN), specificity (SPE), balanced accuracy (BAC), positive predicted value (PPV), negative predictive value (NPV), F1-Score (F1), and area under the receiver operating characteristic curve (AUC).

### 4.2. Methods for Comparison

In this work, we compare the proposed AMNI method with six traditional machine learning methods and three popular deep learning methods. More details can be found below.

(1) **PCA+SVM-s**: The PCA+SVM-s method only uses sMRI data. The 3D image of the whole brain is down-sampled from 121 × 145 × 121 to 61 × 73 × 61, and further flattened into a vectorized feature representation for each subject. We use principal component analysis (PCA) (Wold et al., 1987) by keeping the top 32 principal components to reduce feature dimension based on the above feature representations of all subjects. Finally, the support vector machine (SVM) with Radial Basis Function (RBF) kernel is employed for classification.

(2) **EC+SVM**: The EC+SVM method uses rs-fMRI data. Similar to our AMNI, we first construct a functional connectivity matrix based on Pearson correlation coefficient for each subject. We then extract eigenvector centralities (EC) (Bonacich, 2007), which measure a node's importance while giving consideration to the importance of its neighbors in the FC network, as features of the FCN and feed these 112-dimensional features into an SVM classifier with RBF kernel for disease detection.

(3) **DC+SVM**: Similar to EC+SVM, the DC+SVM method first constructs a FCN based on Pearson correlation coefficient for each subject, and then extracts degree centrality (DC) (Nieminen, 1974) as FCN features by measuring node importance based on the number of links incident upon a node. The 112-dimensional DC features are finally feed into an SVM for classification.

(4) **CC+SVM**: Similar to EC/CC+SVM, this method extracts the local clustering coefficient (CC) (Wee et al., 2012) to measure clustering degree of each node in each FCN. The 112-dimensional CC features are fed into an SVM for classification.

(5) **PCA+SVM-f**: In the PCA+SVM-f method, the upper triangle of a FC matrix is flattened into a vector for each subject after the FC matrix is constructed. Then, we use PCA by keeping the top 32 principal components to reduce feature dimension based on the above feature representations of all subjects. Finally, an SVM is used for classification.

(6) **PP+SVM**: In this method, we integrate rs-fMRI and sMRI features for classification based on SVM. Specifically, we first employ PCA+SVM-s and PCA+SVM-f to extract features from structural and functional MRIs, respectively. Then, we concatenate features of these two modalities for the same subject, followed by an SVM for classification.

(7) **2DCNN**: In this method, we employ the original FC matrix of each subject as input of a CNN model (LeCun et al., 1989). Specifically, this CNN contains three convolutional layers and two fully-connected layers. Each convolutional layer is followed by batch normalization and ReLU activation. The channel numbers for the three convolutional layers are 4, 8, and 8, respectively, and the corresponding size of the convolution kernel is 3 × 3, 5 × 5, 7 × × 7, respectively. The two fully-connected (FC) layers contain 4, 096 and 2 neurons, respectively.

(8) **ST-GCN**: We also compare our method with the spatio-temporal graph convolutional network (ST-GCN), a state-of-the-art method for modeling spatio-temporal dependency of fMRI data (Gadgil et al., 2020). Specifically, the ST-GCN comprises two layers of spatio-temporal graph convolution (ST-GC) units, global average pooling and a fully connected layer. Note that each ST-GC layer produces 64-channel outputs with the temporal kernel size of 11, a stride of 1, and a dropout rate of 0.5.

(9) **3DCNN+2DCNN**: In this method, we employ 3DCNN and 2DCNN to extract features from sMRI and fMRI, respectively. We then concatenate features learned from 3DCNN and 2DCNN, and feed the concatenated features to a fully-connected layer and the softmax layer for classification.

### 4.3. Experimental Results

**Figure 2:**
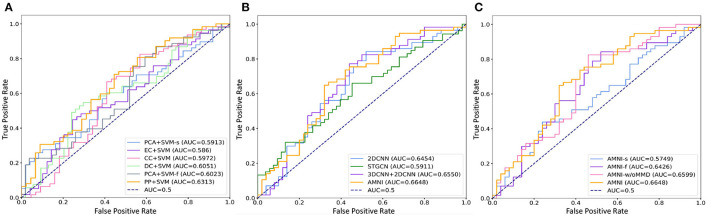
ROC curves and related AUC values achieved by different methods in MDD vs. HC classification. **(A)** AMNI vs. six conventional methods. **(B)** AMNI vs. three deep learning methods. **(C)** AMNI vs. its three variants.

The quantitative results of the proposed AMNI and nine competing methods in the task of MDD vs. HC classification are reported in Table 3. In Figures 2A,B, we also show ROC curves of different methods. From Table 3 and Figures 2A,B, we have the following interesting observations.

**Table 3:** Classification results in terms of “mean (standard deviation)” achieved by ten methods in MDD vs. HC classification, with best results shown in bold.

**Method**	**Data**	**ACC**	**SEN**	**SPE**	**BAC**	**PPV**	**NPV**	**F1**	**AUC**
PCA+SVM-s	S	0.566 (0.011)	0.669 (0.021)	0.456 (0.007)	0.563 (0.010)	0.580 (0.006)	0.553 (0.017)	0.618 (0.013)	0.591 (0.008)
EC+SVM	F	0.560 (0.014)	0.651 (0.009)	0.462 (0.029)	0.557 (0.015)	0.577 (0.013)	0.539 (0.018)	0.609 (0.009)	0.586 (0.019)
CC+SVM	F	0.574 (0.007)	0.674 (0.018)	0.470 (0.014)	0.572 (0.006)	0.589 (0.005)	0.562 (0.011)	0.625(0.009)	0.597(0.014)
DC+SVM	F	0.578 (0.014)	0.676 (0.019)	0.477 (0.016)	0.577 (0.017)	0.593 (0.015)	0.568 (0.021)	0.627 (0.014)	0.605 (0.015)
PCA+SVM-f	F	0.570 (0.011)	0.653 (0.014)	0.483 (0.019)	0.568 (0.012)	0.588 (0.010)	0.554 (0.016)	0.614 (0.009)	0.602 (0.013)
PP+SVM	SF	0.593 (0.026)	0.675 (0.022)	0.502 (0.036)	0.588 (0.027)	0.605 (0.026)	0.578 (0.030)	0.636 (0.022)	0.631 (0.027)
2DCNN	F	0.613 (0.013)	0.670 (0.022)	0.551 (0.024)	0.611 (0.013)	0.628 (0.013)	0.599 (0.016)	0.643 (0.014)	0.645 (0.013)
STGCN	F	0.583(0.022)	0.616 (0.027)	0.544 (0.026)	0.580 (0.022)	0.612 (0.015)	0.548 (0.037)	0.614 (0.018)	0.591 (0.008)
3D+2DCNN	SF	0.632 (0.028)	0.667 (0.022)	0.593 (0.043)	0.630 (0.029)	**0.649 (0.034)**	0.617(0.041)	0.656 (0.026)	0.655 (0.013)
AMNI (Ours)	SF	**0.650 (0.016)**	**0.694 (0.068)**	**0.609 (0.056)**	**0.651 (0.016)**	0.640 (0.031)	**0.667 (0.055)**	**0.663 (0.021)**	**0.665 (0.017)**

*S, sMRI; F, fMRI; SF, sMRI+fMRI*.

*First*, our AMNI and two deep learning methods (i.e., 2DCNN and 3DCNN+2DCNN) generally achieve better performance in terms of eight metrics, compared with six traditional machine learning methods. For example, in terms of ACC values, the AMNI yields the performance improvement of 5.7%, compared with the best traditional machine learning method (e.g., PP+SVM) in MDD detection. These results demonstrate that, deep learning methods that can learn diagnosis-oriented neuroimage features is more effective in MDD detection, compared with traditional machine learning methods that rely on handcrafted features. *Second*, three multimodal methods (i.e., PP+SVM, 3DCNN+2DCNN, and AMNI) generally outperform their single-modality counterparts (i.e., PCA+SVM-s, PCA+SVM-f, and 2DCNN). For instance, both our AMNI and 3DCNN+2DCNN methods that integrate sMRI and fMRI data are superior to 2DCNN which only use functional data. This implies that taking advantage of multimodal MRIs (as we do in this work) helps promote the diagnosis performance, thanks to the complementary information provided by functional and structural MRIs. Furthermore, our proposed AMNI achieves better performance in terms of most metrics, compared with eight competing methods. These results imply that adaptive integration of multimodal neuroimages helps boost the performance of MDD identification.

### 4.4. Statistical Significance Analysis

We further calculate predicted probability distribution difference on test data between our model and each of eight competing methods by paired sample *t*-test. Denote *u*_1_ and *u*_2_ as the population mean of predicted probability distributions from our AMNI and one competing method, respectively. The hypotheses can be expressed as follows:


(12) $$\begin{array}{l} \begin{array}{c} H_{0}:u_{1}=u_{2}\\ H_{1}:u_{1}\neq u_{2} \end{array} \end{array}$$

where *H*_0_ is the null hypothesis, meaning that our model and the competing method do not have significant difference. And *H*_1_ is the alternative hypothesis, meaning that our model and the competing method have significance difference. The test statistic for the paired samples *t*-test is as follows:


(13) $$\begin{array}{l} t=\frac{{\bar{x}}_{\text{diff}}}{s_{\text{diff}}/\sqrt{n}} \end{array}$$

where ${\bar{x}}_{\text{diff}}$ is sample mean of the differences, *s*_diff_ is sample standard deviation of the differences and *n* is the sample size (i.e., number of pairs). The *p*-values that corresponds to the test statistic *t* are shown in Table 4.

**Table 4:** Results of statistical significance analysis between the proposed AMNI and eight competing methods.

**Pairwise comparison**	**p-value**	**p <0.05**
AMNI vs. PCA+SVM-s	3.40 ×10^−4^	Yes
AMNI vs. EC+SVM	3.93 ×10^−4^	Yes
AMNI vs. CC+SVM	3.16 ×10^−4^	Yes
AMNI vs. DC+SVM	2.43 ×10^−4^	Yes
AMNI vs. PCA+SVM-f	1.01 ×10^−5^	Yes
AMNI vs. PP+SVM	2.71 ×10^−5^	Yes
AMNI vs. 2DCNN	9.48 ×10^−3^	Yes
AMNI vs. 3DCNN+2DCNN	1.07 ×10^−3^	Yes

As shown in Table 4, all obtained *p*-values are less than our chosen significance level (i.e., 0.05). Therefore, *H*_0_ is rejected, which means that our AMNI method differs significantly from each of the eight competing methods.

### 4.5. Feature Visualization

**Figure 3:**
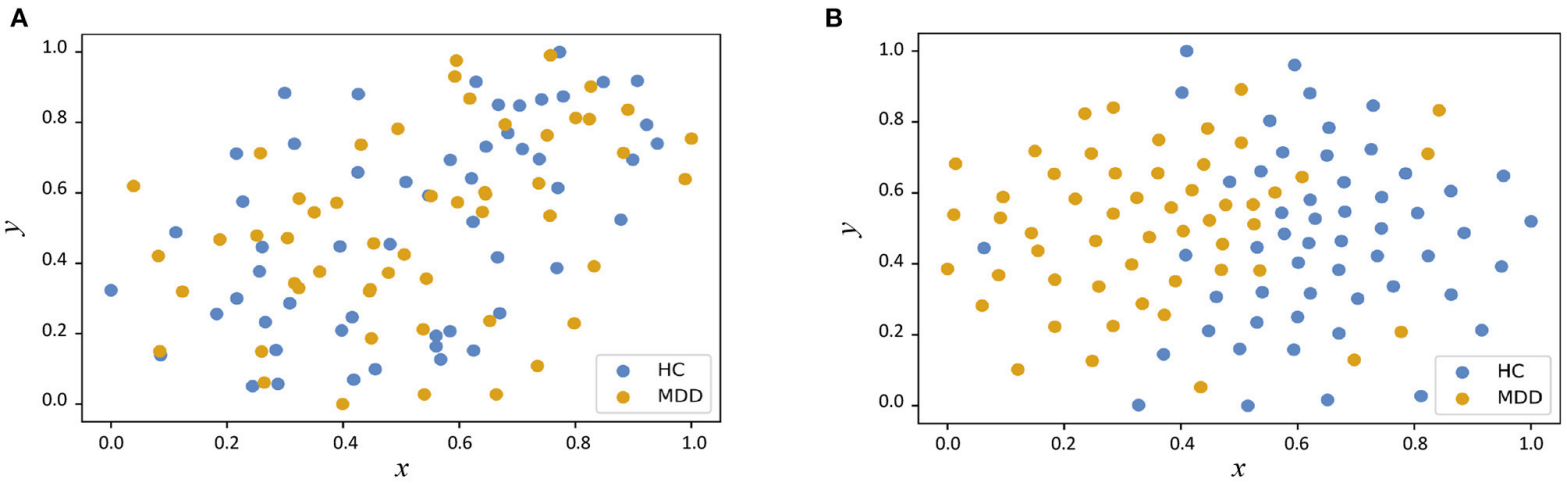
Visualization of feature distributions from the PP+SVM and the proposed AMNI models via t-SNE (Van der Maaten and Hinton, 2008). The horizontal and vertical axes denote two dimensions after feature mapping. **(A)** Distribution of features derived from PP+SVM. **(B)** Distribution of features derived from AMNI.

In Figure 3, we visualize the data distributions of features derived from two multimodal methods (i.e., PP+SVM and AMNI) *via* t-SNE (Van der Maaten and Hinton, 2008). Note that the features of PP+SVM are generated by concatenating handcrafted features from two modalities, while the features of our AMNI are extracted based on an end-to-end deep learning model (see Figure 1). As shown in Figure 3, the feature distributions of two categories (i.e., MDD and HC) generated from our AMNI method have more significant difference, while their feature distribution gap is not evident for the PP+SVM method. This may indicate that our AMNI can learn more discriminative features for MDD detection by explicitly reducing the inter-modality discrepancy, compared with the traditional PP+SVM method.

## 5. Discussion

### 5.1. Ablation Study

**Figure 4:**
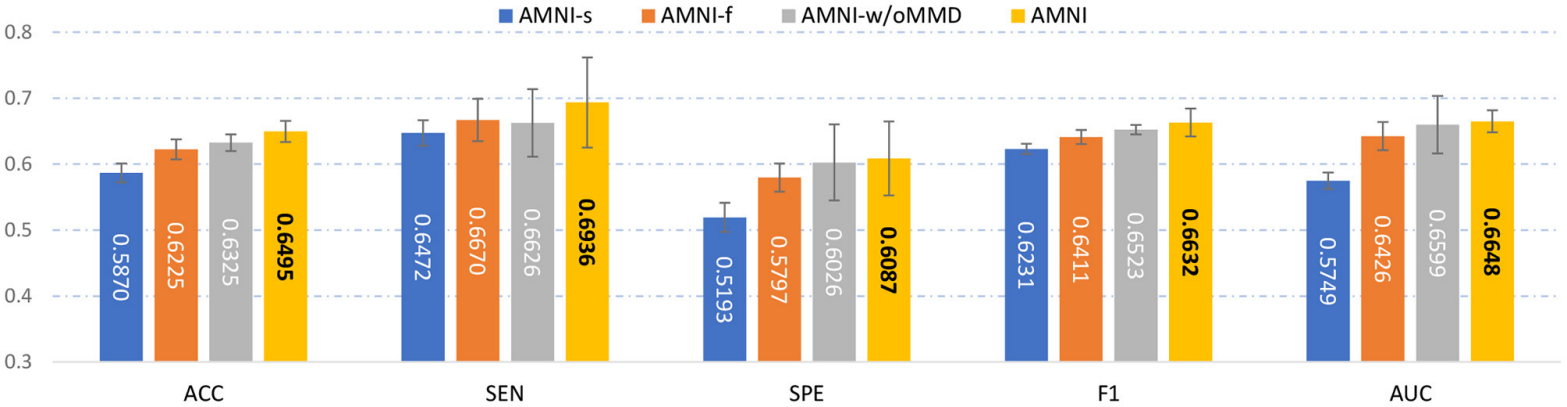
Performance of our AMNI and its three variants in the task of MDD vs. HC classification, with best results shown in bold.

To evaluate the effectiveness of each component in the proposed AMNI, we further compare AMNI with its three variants: (1) **AMNI-s** that only uses CNN branch and feature fusion module of AMNI, without considering functional MRI, (2) **AMNI-f** that only uses GCN branch and feature fusion module of AMNI, without considering structural MRI, (3) **AMNI-w/oMMD** that directly feeds concatenated fMRI and sMRI features (*via* GCN and CNN modules, respectively) into the feature fusion module for classification, without using the proposed feature adaption module. The experimental results are reported in Figures 4, 2C.

It can be seen from Figure 4 that two multimodal methods (i.e., AMNI-w/oMMD and AMNI) generally outperform the single modality methods (i.e., AMNI-s and AMNI-f). This further demonstrates that multimodal data can provide complementary information to help boost the performance of MDD identification. Besides, our AMNI achieves consistently better performance compared with AMNI-w/oMMD that ignores the heterogeneity between the two modalities. These results further validate the effectiveness of the proposed feature adaption module in alleviating the inter-modality discrepancy between different modalities. In addition, Figure 2C suggests that our proposed AMNI achieves good ROC performance and the best AUC value compared with its three variants.

### 5.2. Influence of Hyperparameter

**Figure 5:**
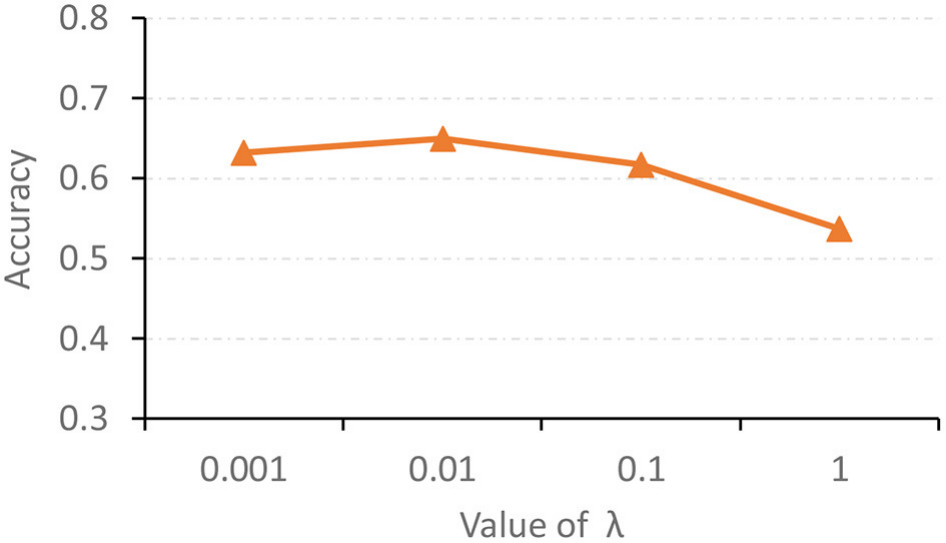
Accuracy achieved by the proposed AMNI method with different values of λ in Equation (11) in the task of MDD vs. HC classification.

The hyperparameter λ in Equation (11) is used to tune the contribution of the proposed feature adaptation module for re-calibrating feature distributions of two modalities. We now report the classification accuracy of the proposed AMNI with different values of λ in Figure 5. As shown in Figure 5, with λ = 0.01, our AMNI can achieve best performance. But using a too large value (e.g., λ = 1) will yield worse performance. A possible reason is that focusing too much on the reduction of differences between modalities (with a large λ) may lose the specific and unique information of each modality, thereby degrading the learning performance.

### 5.3. Influence of Graph Construction Strategy

**Figure 6:**
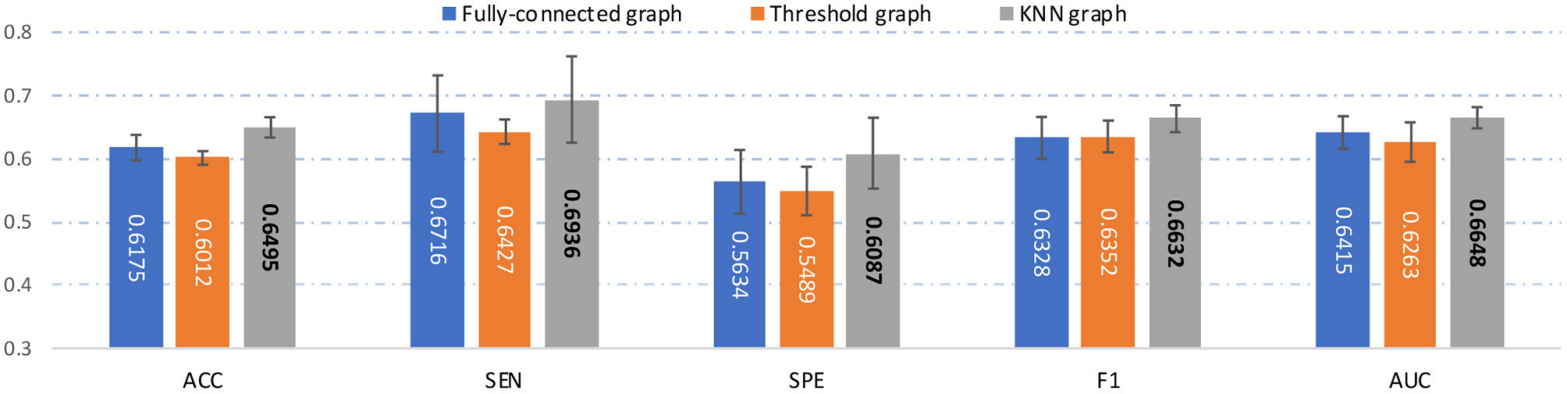
Results of the proposed AMNI based on three different graph construction methods (e.g.fully-connected graph, threshold graph, and KNN graph) in the task of MDD vs. HC classification, with best results shown in bold.

In the main experiment, we build a KNN graph to generate an adjacency matrix for each FCN. To investigate the influence of the use of different graph construction strategies, besides KNN, we also construct a fully-connected graph and a threshold graph to generate the adjacency matrix, respectively. For the fully-connected graph, we directly take *A* = (|*w*_*ij*_|) as the adjacency matrix, which is an edge-weighted graph. For the threshold graph, we generate the adjacency matrix *A* by binarizing the FC matrix *B* to regulate the sparsity of the graph. Thus, the adjacency matrix can be described as $A=\left(a_{ij}\right)\in {\left\{0,1\right\}}^{N\times N}$, where *a*_*ij*_ = 1 if the connection coefficient between *i*-th and *j*-th ROI is greater than a threshold *q*; and *a*_*ij*_ = 0, otherwise. The threshold *q* is set as 0.2 here. The experimental results of our AMNI with three different graph construction strategies are reported in Figure 6.

As can be seen from Figure 6, our AMNI model based on KNN graph outperforms its two variants that use fully-connected graph and threshold graph. The underlying reason could be that KNN graph can preserve node-centralized local topology information while removing noisy/redundant information in graph (Ktena et al., 2018; Yao et al., 2021).

### 5.4. Influence of Network Architecture

To explore the influence of different network architectures of AMNI on the experimental results, we adjust the the network depth of two branches of the AMNI model, respectively. *On the one hand*, with the CNN branch fixed, we vary the number of graph convolutional layers for the GCN branch of AMNI and report the corresponding results of AMNI in Table 5. This table shows that the AMNI achieves the overall best performances (e.g., ACC=0.6495 and AUC=0.6648) with two graph convolutional layers in the GCN branch. In addition, as the number of graph convolutional layers increases (see AMNI-G3 and AMNI-G4), the performance is not good. This may be due to the over-smoothing problem (that is, Laplacian smoothing makes the node representations more similar as the graph convolutional layer increases; Yang et al., 2020), which may reduce the discriminative compatibility of learned features. *On the other hand*, we fix the GCN branch and vary the architecture of the CNN in AMNI for performance evaluation. Specifically, we vary the number of convoluational layers in CNN within [3, 6] and report the results of AMIN in MDD vs. HC classification in Table 5. This table shows that fine-tuning the network architecture of the CNN branch in AMNI achieves comparable results, which implies that our AMNI is robust to different network architectures. Further, AMNI with five convoluational layers in the CNN branch (e.g., AMNI-G5) achieves better performance in terms of accuracy, sensitivity, balanced accuracy, positive predicted value and F1-Score.

**Table 5:** Classification results of our AMNI in MDD vs. HC classification with different network depth, with best results shown in bold.

**Method**	**ACC**	**SEN**	**SPE**	**BAC**	**PPV**	**NPV**	**F1**	**AUC**
AMNI-G1	0.634 (0.014)	0.677 (0.065)	0.587 (0.054)	0.632 (0.019)	**0.669 (0.041)**	0.598 (0.077)	**0.669 (0.011)**	0.627 (0.032)
AMNI-G2	**0.650 (0.016)**	**0.694 (0.068)**	**0.609 (0.056)**	**0.651(0.016)**	0.640 (0.031)	**0.667 (0.055)**	0.663 (0.021)	**0.665 (0.017)**
AMNI-G3	0.595 (0.008)	0.629 (0.034)	0.559 (0.041)	0.594 (0.010)	0.600 (0.010)	0.590 (0.019)	0.614 (0.016)	0.605 (0.009)
AMNI-G4	0.587 (0.011)	0.618 (0.023)	0.554 (0.025)	0.586 (0.011)	0.610 (0.042)	0.561 (0.036)	0.613 (0.022)	0.599 (0.022)
AMNI-C3	0.628 (0.005)	0.692 (0.045)	0.551 (0.057)	0.622 (0.007)	0.647 (0.014)	0.603 (0.012)	0.668 (0.013)	0.622 (0.007)
AMNI-C4	0.650 (0.016)	0.694 (0.068)	**0.609 (0.056)**	0.651 (0.016)	0.640 (0.031)	**0.667 (0.055)**	0.663 (0.021)	**0.665 (0.017)**
AMNI-C5	**0.660 (0.022)**	**0.742 (0.042)**	0.565 (0.049)	**0.653 (0.023)**	**0.663 (0.011)**	0.657 (0.040)	**0.700 (0.020)**	0.653 (0.023)
AMNI-C6	0.642 (0.014)	0.701 (0.046)	0.580 (0.041)	0.641 (0.017)	0.651 (0.029)	0.634 (0.053)	0.673 (0.008)	0.628 (0.018)

*Note that AMNI-Gn contains n graph convolutional layers in the GCN module of AMNI, and AMNI-Cn contains n convolutional layers in the CNN module of AMNI*.

Besides, we also further discuss the influence of network width of each branch on the experimental results. *For one thing*, with the CNN branch fixed, we change the number of neurons in the graph convolutional layers and then report the corresponding results of AMNI in Table 6. It can be found from Table 6 that the AMINI model using different numbers of neurons in graph convolutional layers achieves comparable experimental results, which means our model is not very sensitive to the change of network width of the GCN branch. *For another thing*, with the GCN branch fixed, we change the number of filters in each 3D convolutional layer and record the results in Table 6. As shown in Table 6, with the increase of the number of filters in 3D CNN module of AMNI, the model (i.e., AMNI-c3 and AMNI-c4) generally achieves better performance. This may be due to that using more filters in CNN can capture richer features across global and local information of sMRI.

**Table 6:** Classification results of our AMNI in MDD vs. HC classification with different network width, with best results shown in bold.

**Method**	**ACC**	**SEN**	**SPE**	**BAC**	**PPV**	**NPV**	**F1**	**AUC**
AMNI-g40	0.620 (0.035)	0.626 (0.089)	0.614 (0.097)	0.620 (0.035)	0.652 (0.039)	0.593 (0.040)	0.635 (0.049)	0.650 (0.036)
AMNI-g64	**0.650 (0.016)**	0.694 (0.068)	0.609 (0.056)	**0.651 (0.016)**	0.640 (0.031)	**0.667 (0.055)**	0.663 (0.021)	0.665 (0.017)
AMNI-g88	0.626 (0.015)	**0.697 (0.048)**	0.542 (0.052)	0.620 (0.015)	0.644 (0.016)	0.604 (0.023)	**0.669 (0.021)**	**0.667 (0.011)**
AMNI-g112	0.631 (0.016)	0.647 (0.053)	**0.612 (0.037)**	0.629 (0.015)	**0.659 (0.015)**	0.602 (0.024)	0.651 (0.029)	0.637 (0.037)
AMNI-c1	0.598 (0.017)	0.643 (0.046)	0.535 (0.081)	0.589 (0.028)	**0.643 (0.028)**	0.535 (0.073)	0.642 (0.026)	0.607 (0.0148)
AMNI-c2	0.630 (0.020)	0.693 (0.080)	0.575 (0.096)	0.634 (0.016)	0.593 (0.033)	**0.685 (0.029)**	0.635 (0.023)	0.667 (0.004)
AMNI-c3	**0.650 (0.016)**	**0.694 (0.068)**	0.609 (0.056)	**0.651 (0.016)**	0.640 (0.031)	0.667 (0.055)	**0.663 (0.021)**	0.665 (0.017)
AMNI-c4	0.641 (0.015)	0.654 (0.051)	**0.629 (0.030)**	0.642 (0.015)	0.628 (0.030)	0.658 (0.044)	0.638 (0.028)	**0.689 (0.042)**

*Note that AMNI-gn contains n neurons in the graph convolutional layers of the GCN module. And the filter sequences in CNN module of AMNI-c1, AMNI-c2, AMNI-c3 and AMNI-c4 are [4, 8, 16, 32], [8, 16, 32, 64], [16, 32, 64, 128], and [32, 64, 128, 256], respectively*.

### 5.5. Influence of Multimodality Fusion Strategy

**Figure 7:**
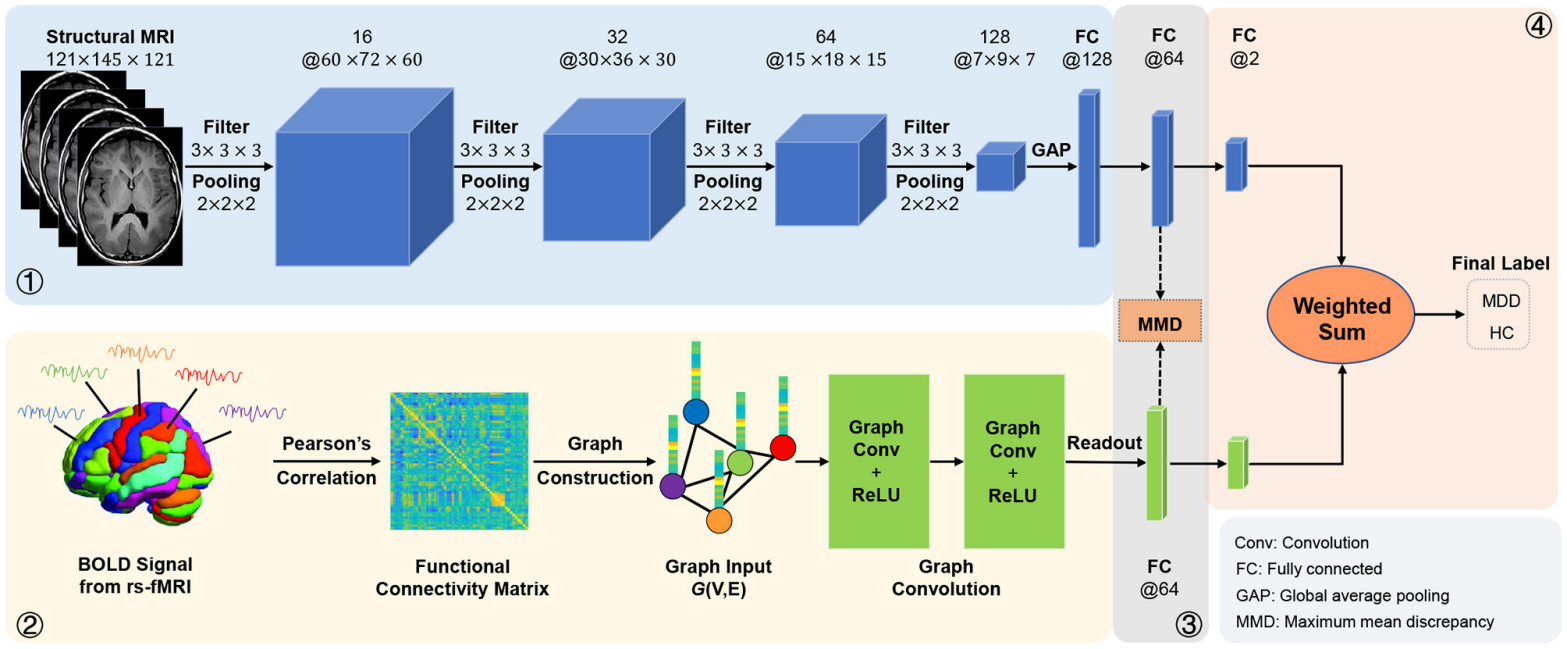
Illustration of the adaptive multimodal neuroimage integration (AMNI) framework based on a decision-level fusion strategy.

**Figure 8:**
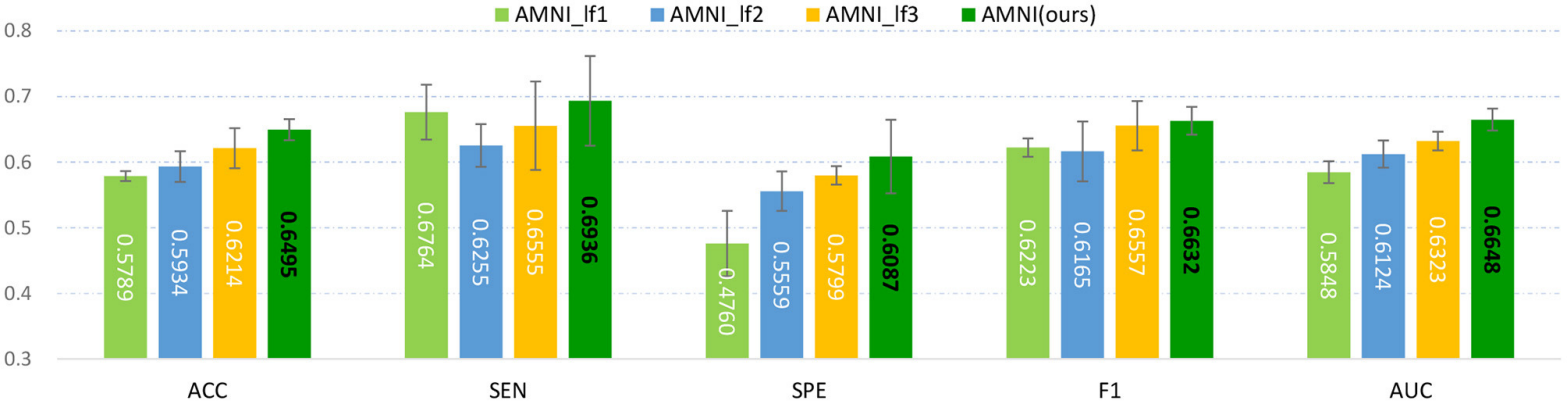
Experimental results of late fusion method and our AMNI method in MDD vs. HC classification. Note that AMNI_lf1, AMNI_lf2, and AMNI_lf3 denote that the weight ratio between fMRI and sMRI branch is $\frac{0.2}{0.8}$, $\frac{0.5}{0.5}$, and $\frac{0.8}{0.2}$, respectively.

We fuse fMRI and sMRI data at the feature-level (see Figure 1) in the main experiments. We further investigate the influence of different fusion strategies by comparing our AMNI (using feature-level fusion) with its variant (called **AMNI_lf**) using a decision-level fusion strategy. As shown in Figure 7, in the AMNI_lf, the fMRI feature derived from GCN is fed into two fully connected layers and a Softmax layer for feature abstraction and classification. Similarly, the sMRI feature derived from CNN is fed into three fully connected layers and a Softmax layer. The outputs of these two branches are further fused *via* a weighted sum operation. We vary the weighted ratio between fMRI and sMRI branches within [$\frac{0.2}{0.8}$, $\frac{0.5}{0.5}$, $\frac{0.8}{0.2}$] and denote these three methods as AMNI_lf1, AMNI_lf2, and AMNI_lf3, respectively, with the experimental results shown in Figure 8.

As shown in Figure 8, as the weight of GCN branch increases, the model achieves better performance in terms of most metrics. However, the results of AMNI using the decision-level fusion method are generally inferior to that of the feature-level fusion method proposed by this article. This implies that feature-level fusion of functional and structural representations could be more effective.

### 5.6. Limitations and Future Work

Several limitations need to be considered. First, we only integrate T1-weighted MRI and functional MRI data for automated MDD diagnosis. Actually, diffusion tensor imaging (DTI) data can examine and quantify white matter microstructure of the brain, which can further help uncover the neurobiological mechanisms of MDD. Therefore, it is valuable to incorporate DTI data into multimodal research in our future work. Second, we use functional connectivity networks for representing rs-fMRI data and treat them as input of the proposed method. It is interesting to extract diagnosis-oriented fMRI features, as we do for T1-weighed MRIs, which will also be our future work. Besides, a feature adaptation module with a cross-modal MDD loss is designed for reducing inter-modality data heterogeneity. Many other data adaptation methods (Ben-David et al., 2007) can also be incorporated into the proposed AMNI framework for further performance improvement.

## 6. Conclusion

In this article, we propose an adaptive multimodal neuroimage integration (AMNI) framework for automated MDD diagnosis based on functional and structural MRI data. We first employ GCN and CNN to learn feature representations of functional connectivity networks and structural MR images. Then, a feature adaptation module is designed to alleviate inter-modality difference by minimizing the distribution difference between two modalities. Finally, high-level features extracted from functional and structural MRI modalities are integrated and delivered to a classifier for disease detection. Experimental results on 533 subjects with rs-fMRI and T1-weighted sMRI demonstrate the effectiveness of the proposed method in identifying MDD patients from healthy controls.

## Data Availability Statement

The datasets presented in this study can be found in online repositories. The names of the repository/repositories and accession number(s) can be found at: REST-meta-MDD Consortium Data Sharing.

## Ethics Statement

The studies involving human participants were reviewed and approved by REST-meta-MDD Consortium Data Sharing. The patients/participants provided their written informed consent to participate in this study.

## Author Contributions

QW and ML designed the study. QW downloaded and analyzed the data, performed experiments, and drafted the manuscript. QW, LL, LQ, and ML revised the manuscript. All authors read and approved the final manuscript.

## Funding

QW and LQ were partly supported by National Natural Science Foundation of China (Nos. 62176112, 61976110, and 11931008), Natural Science Foundation of Shandong Province (Nos. ZR2018MF020 and ZR2019YQ27), and Taishan Scholar Program of Shandong Province.

## Conflict of Interest

The authors declare that the research was conducted in the absence of any commercial or financial relationships that could be construed as a potential conflict of interest.

## Publisher's Note

All claims expressed in this article are solely those of the authors and do not necessarily represent those of their affiliated organizations, or those of the publisher, the editors and the reviewers. Any product that may be evaluated in this article, or claim that may be made by its manufacturer, is not guaranteed or endorsed by the publisher.

## Footnotes

^1^ http://rfmri.org/REST-meta-MDD
